# Macrophages in psoriasis: a bibliometric analysis of research trends, knowledge gaps, and future directions (2015–2024)

**DOI:** 10.3389/fmed.2026.1737689

**Published:** 2026-04-15

**Authors:** Hongda Yu, Yifei Wang, Qiuchen Huang, Linghan Meng, Jingna Tao, Yanping Bai

**Affiliations:** 1 Department of Dermatology, China National Center for Integrated Traditional Chinese and Western Medicine, China-Japan Friendship Hospital, Beijing, China; 2 Department of Anorectum, Dongzhimen Hospital, Beijing, China; 3 Graduate School, Beijing University of Chinese Medicine, Beijing, China

**Keywords:** bibliometric analysis, immunity, macrophage, psoriasis, research trends

## Abstract

**Objective:**

To systematically analyze the research landscape, knowledge structure, and evolution hotspots in macrophages and psoriasis research over the past decade through bibliometric analysis, providing guidance for mechanistic investigation and therapeutic strategies.

**Methods:**

Literature published between January 2015 and December 2024 was retrieved from the Web of Science database and Scopus. The softwares CiteSpace, VOSviewer, Scimago Graphica, and Excel were employed for bibliometric analysis, including co-occurrence networks, clustering analysis, and keyword evolution analysis.

**Results:**

A total of 2012 articles were identified from 2015 to 2024, showing an upward trend first and then slightly decreased. China and the United States were the leading contributors, while international collaboration formed four major clusters. Core journals such as *Frontiers in Immunology* and *Journal of Immunology* played pivotal roles in knowledge dissemination. Research hotspots evolved from fundamental inflammatory mechanisms to targeted biologic therapies, highlighting a growing emphasis on precision medicine and the immunopathogenesis of psoriasis.

**Conclusion:**

Macrophage and psoriasis-related research represents a rapidly evolving interdisciplinary field transitioning from isolated pathological studies to comprehensive research integrating advanced technologies with clinical applications. While substantial progress has been achieved in exploring macrophage roles in psoriasis pathogenesis, significant gaps remain between laboratory discoveries and clinical translation. Future research should prioritize international collaboration, application studies, and precision therapeutic strategies targeting macrophage-mediated pathways.

## 1 Introduction

Psoriasis is a chronic, recurrent, inflammatory autoimmune disease characterized by abnormal proliferation of keratinocytes and dysregulation of the immune system (1, 2). Psoriasis encompasses multiple clinical phenotypes, with plaque psoriasis (psoriasis vulgaris) representing the most prevalent form affecting approximately 80–90% of patients, while pustular psoriasis constitutes a rarer but more severe variant characterized by sterile neutrophil-filled pustules. Although both forms involve immune dysregulation, macrophages appear to play a more prominent role in plaque psoriasis pathogenesis. In plaque-type lesions, macrophages constitute a major cellular infiltrate in the dermis and contribute significantly to TNF-α, IL-23, and IL-1β production, driving the Th17-mediated inflammatory cascade. In contrast, pustular psoriasis demonstrates stronger associations with IL-36 pathway dysregulation and neutrophil-dominated pathology, with relatively less evidence for macrophage centrality. Epidemiological studies indicate that psoriasis affects approximately 125 million people worldwide, posing a significant health and economic burden to both patients and society (3, 30). The characteristic skin lesions of this disease are well-demarcated red papules or plaques covered with multiple layers of silvery-white scales, accompanied by clinical symptoms such as pruritus. The characteristic pathological manifestations include hyperproliferation and abnormal differentiation of epidermal keratinocytes, dilation of capillaries in the dermal papillary layer, as well as inflammatory cell infiltration (4). According to conventional theory, psoriasis is primarily driven by adaptive immune dysfunction, particularly involving T lymphocytes. Consequently, suppressing cytokine-mediated autoimmune responses has played a critical role in the treatment of psoriasis. However, recent studies have revealed that the innate immune system, especially macrophages, plays a significant role in the initiation, maintenance, and resolution of the disease. The interplay between adaptive and innate immunity influences the pathological progression of psoriasis (5, 6).

Macrophages are one of the core components of the human innate immune system. They are primarily derived from monocytes in the blood and are involved in various innate immune processes (7). Simultaneously, macrophages exhibit remarkable plasticity and heterogeneity. They can differentiate into functionally distinct subsets in response to different microenvironmental signals. Based on their activation states and functional characteristics, macrophages are primarily categorized into classically activated M1 type (pro-inflammatory phenotype) and alternatively activated M2 type (anti-inflammatory phenotype) (8, 9). During the active phase of psoriasis, M1-type macrophages predominate. They secrete large quantities of pro-inflammatory cytokines such as Tumor Necrosis Factor (TNF)-α, Interleukin (IL)-1β, IL-6, and IL-23, which amplify inflammatory cascades and directly contribute to the abnormal proliferation and differentiation of keratinocytes. Meanwhile, M2-type macrophages play a protective role during disease resolution by limiting excessive tissue damage and promoting tissue repair. Furthermore, macrophages interact closely with other immune cells, influencing the differentiation and migration of T cells and thereby contributing to the pathogenesis and progression of psoriasis (10). In summary, macrophages play a central regulatory role in both the initiation of inflammation and the process of tissue repair in psoriasis through their plasticity and the balance between M1 and M2 phenotypes, making them a potential therapeutic target.

Bibliometrics, which involves the quantitative analysis of the structure, distribution, and evolution of academic literature, can objectively reveal the development trajectory of a discipline. With the exponential growth of scientific publications, it has gradually become an important tool for research evaluation, disciplinary planning, and resource allocation (11, 12). Although research on the role of macrophages in psoriasis has grown rapidly, this field still lacks systematic review and synthesis. This study employs bibliometric analysis to systematically examine the knowledge structure, evolving research hotspots, and collaborative networks within psoriasis and macrophage-related research over the past decade. It aims to identify core research groups and highly influential publications, reveal thematic evolution in the field, provide a theoretical map for mechanistic investigation and targeted therapy exploration, and ultimately promote translational clinical research.

## 2 Methods

### 2.1 Data collection and search strategy

The advanced searches were conducted on August 7, 2025 in the Web of Science (WOS) database and Scopus using the following query: TS = (“Psoriasis” OR “psoriatic” OR “Palmoplantaris Pustulosis” OR “Pustulosis Palmaris et Plantaris” OR “Pustulosis of Palms and Soles”) AND TS = (“macrophage^*^”). Only papers published from January 1, 2015 to December 31, 2024 were included. Types of documents like meeting abstracts, conference papers, editorials, book chapters, and retracted papers were excluded. Only English-language Articles and Review Articles were kept. Finally, import the literature obtained from WOS and Scopus into CiteSpace for deduplication and consolidation.

### 2.2 Analytical tools

This study used several tools for analysis. CiteSpace helped create literature co-citation networks and track changes in keywords. VOSviewer was used to build and show collaboration and keyword co-occurrence networks. Scimago Graphica mapped partnerships between countries and regions. Basic number work and graph making were done using WPS Excel and GraphPad Prism.

#### 2.2.1 Bibliometric parameter settings

The analysis in CiteSpace was configured with the following parameters: (1) Time Slicing: from 2015 to 2024, divided into consecutive one-year intervals; (2) Selection Criteria: g-index (k = 10); (3) Network Pruning: the Pathfinder algorithm was applied to prune each time-slice network individually, followed by a second application to simplify the merged overall network; (4) Visualization: node diameter is proportional to frequency of occurrence, and link thickness corresponds to co-occurrence strength.

#### 2.2.2 Collaboration and co-occurrence network analysis

The analytical process in VOSviewer comprises the following steps: (1) Data Preprocessing: the raw data file retrieved from Web of Science was stored in the UTF-8 encoding format; (2) Network Construction and Layout Optimization: the network layout was refined via the Linlog/modularity-based algorithm; (3) Node Weight Assignment: node size bore a proportional relationship to either the number of publications or citations, where the specific weighting standards were defined based on the analysis objectives.

#### 2.2.3 Visualization of national cooperation network

Scimago Graphica was mainly used to analyze and visualize the country collaboration network. The specific procedure was as follows: the country cooperation data saved in GML format from VOSviewer was brought into Scimago Graphica, with parameters configured as follows: (1) node labels were set to “Country”; (2) clustering was based on the country name string. In the network visualization generated from this process, the diameter of each node corresponds to a country's publication output, while the thickness of links denotes the frequency of collaborative efforts among different countries.

#### 2.2.4 Basic statistical analysis and visualization

WPS Excel 2023 and GraphPad Prism 10.1 were primarily employed to perform descriptive statistical analyses and to visualize the resulting data through various chart types, including doughnut charts, bar graphs, and column graphs.

## 3 Results

### 3.1 Annual trends in publications and citations

**Figure 1:**
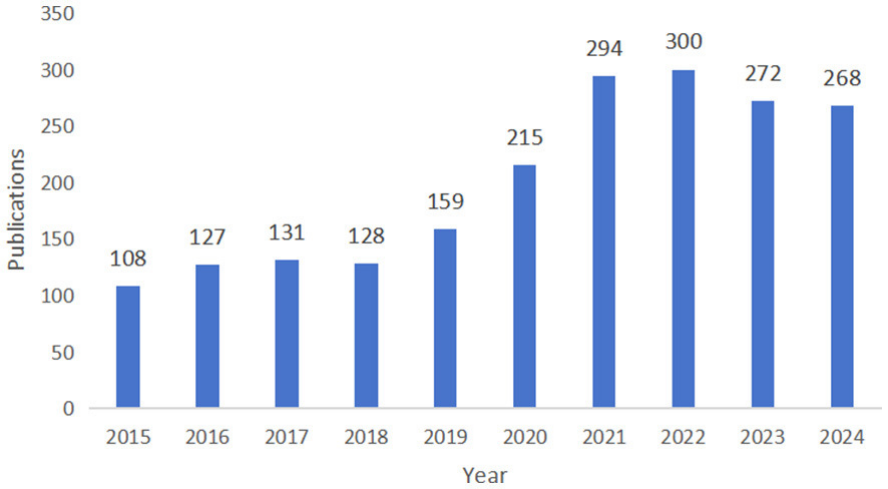
Annual publication volume from 2015 to 2024.

Between 2015 and 2024, a total of 2012 academic articles were published in the field of psoriasis and macrophage-related research, with an average of 201.2 articles per year. The annual publication output exhibited an overall pattern of initial growth followed by a decline: climbing steadily from 108 publications in 2015, peaking at 300 in 2022, before experiencing a modest decline and remaining substantial at 268 by 2024. This reflects the growing academic attention within the field, indicating that the role of macrophages in the pathogenesis of psoriasis is increasingly recognized by the academic community (Figure 1).

### 3.2 National contributions and cross-border research networks

**Figure 2:**
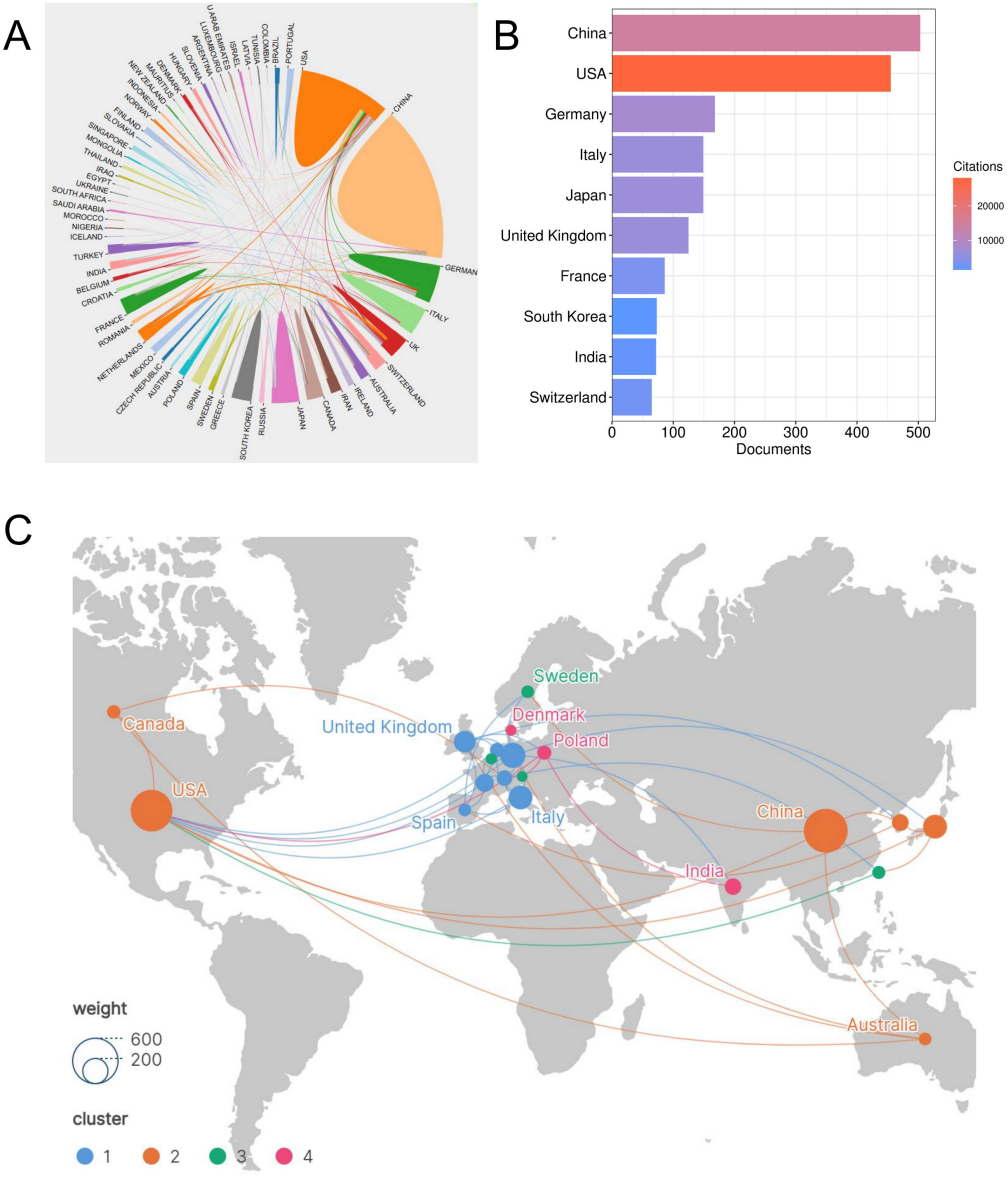
**(A)** International collaboration chord diagram illustrating inter-country linkages. **(B)** The publication counts of the top 10 productive countries (2015–2024). **(C)** Country collaboration network based on co-authorship links. In the international collaboration network, the node size corresponds to the publication output, while the connection width reflects the collaboration intensity.

The field of psoriasis and macrophage research demonstrates a distinctly international research landscape (Figure 2A). The top ten countries by publication output are as follows: China (503 articles), the United States (455 articles), Germany (168 articles), Italy and Japan (both 149 articles), the United Kingdom (125 articles), France (86 articles), South Korea (73 articles), India (72 articles) and Switzerland (65 articles) (Figure 2B, Table 1). Geographically, European countries account for half of the top ten. Asian countries show prominent performance, and North America, represented by the United States, maintains strong research capabilities, reflecting the globalized nature of this field.

**Table 1:** Top 10 contributing countries in psoriasis and macrophage research (2015–2024).

Rank	Country	Documents	Citations	Total link strength	Average citations
1	China	503	14,676	80	29.18
2	USA	455	28,074	155	61.70
3	Germany	168	7,829	158	46.60
4	Italy	149	6,514	82	43.72
5	Japan	149	6,604	46	44.32
6	United Kingdom	125	6,515	71	52.12
7	France	86	3,584	84	41.67
8	South Korea	73	1,447	21	19.82
9	India	72	1,912	16	26.56
10	Switzerland	65	2,859	72	43.98

Analysis of international collaboration networks revealed four major cooperative clusters (Figure 2C): Cluster 1 (blue) consist of European countries such as Netherlands, France, Germany, the United Kingdom, Italy, Spain, and Switzerland; Cluster 2 (orange), with the United States and China as cores, includes Australia, Canada, South Korea, forming an extensive transcontinental collaborative network; Cluster 3 (green) mainly comprises Austria, Sweden and Belgium; Cluster 4 (pink) includes Poland, India and Denmark. It is important to note that the clustering algorithm groups countries based on overall network similarity rather than direct bilateral collaborations. Countries within the same cluster share similar collaboration patterns and network neighborhoods, which may involve indirect connections through common collaborating partners rather than direct co-authorship relationships. For instance, Cluster 4 includes Poland, India, and Denmark, which may not have extensive direct collaborations with each other but share similar collaboration profiles with other countries in the network.

### 3.3 Core journals and citation profile

**Figure 3:**
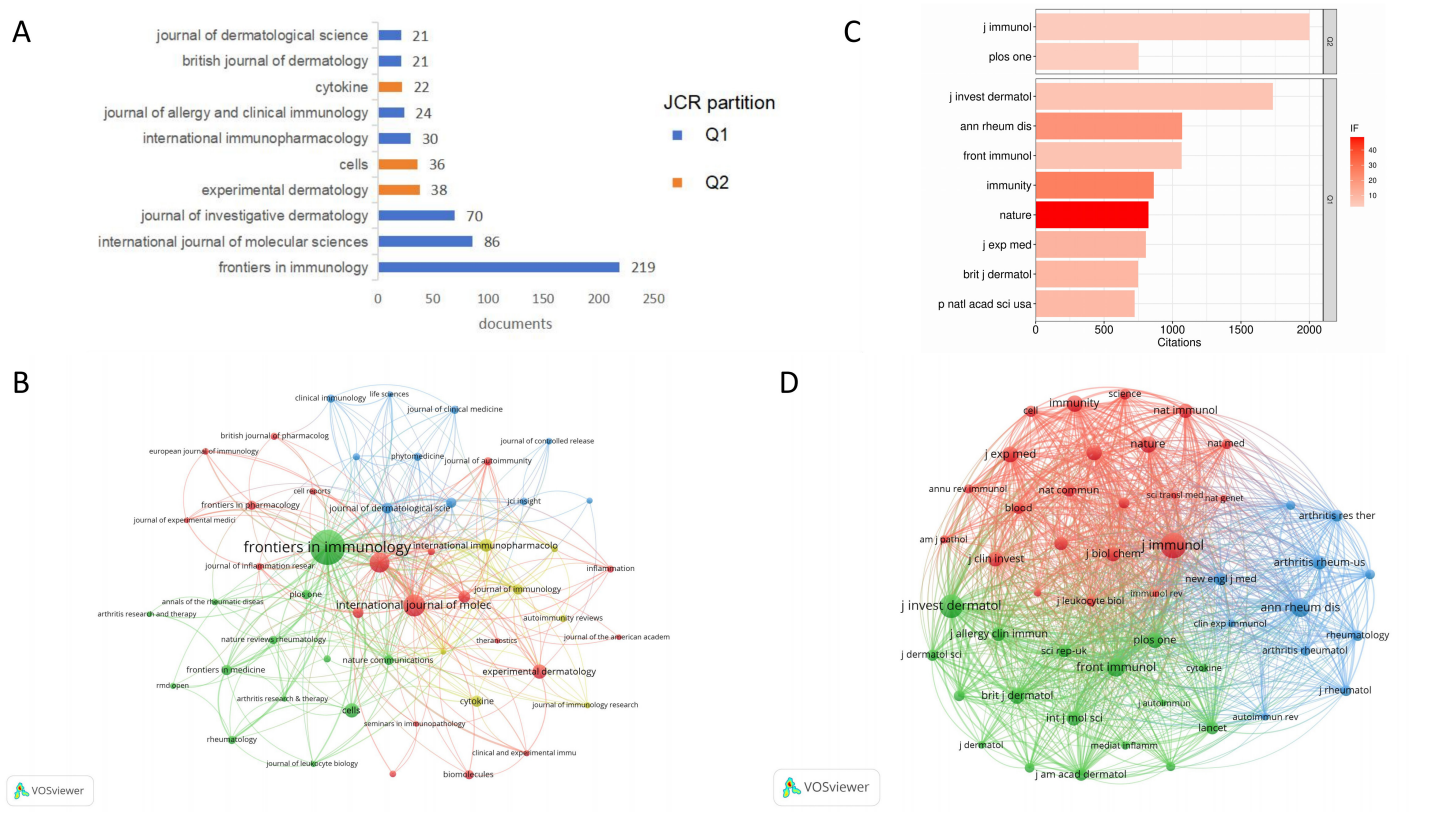
**(A)** The top 10 journals in terms of publication volume. **(B)** Journal co-occurrence network analysis of macrophage and psoriasis publications. **(C)** The top 10 journals by citation count. **(D)** Journal co-citation network analysis of macrophage and psoriasis publications.

In terms of publication volume, the top ten journals in this field are as follows: *Frontiers in Immunology* (219 articles), *International Journal of Molecular Sciences* (86 articles), *Journal of Investigative Dermatology* (70 articles), *Experimental Dermatology* (38 articles), *Cells* (36 articles), *International Immunopharmacology* (30 articles), *Journal of Allergy And Clinical Immunology* (24 articles), *Cytokine* (22 articles), *British Journal of Dermatology* (21 articles), *Journal of Dermatological Science* (21 articles). These journals span multiple disciplines including immunology, dermatology, biomedicine, and pharmacology (Figure 3A). Numerous connecting lines between multidisciplinary journals represent citations and collaborative relationships, reflecting the interdisciplinary nature of research in the psoriasis and macrophage field (Figure 3B). Regarding journal ranking, seven of the top 10 journals rank Q1 in Journal Citation Reports system (JCR), and three rank Q2, indicating that research on this topic is primarily published in high-impact journals. In terms of journal citations, the *Journal of Immunology* and the *Journal of Investigative Dermatology* have the highest citation frequencies, with 2001 and 1733 citations respectively (Figure 3C), demonstrating their significant influence and important role in advancing the field. Although *Nature* is not the most cited journal in this specific area, its high impact factor underscores the cutting-edge nature and importance of the research. The citation network graph shows that the *Journal of Immunology* is located at the core position, reflecting its important position in the field (Figure 3D).

### 3.4 Author influence and collaboration structure

**Figure 4:**
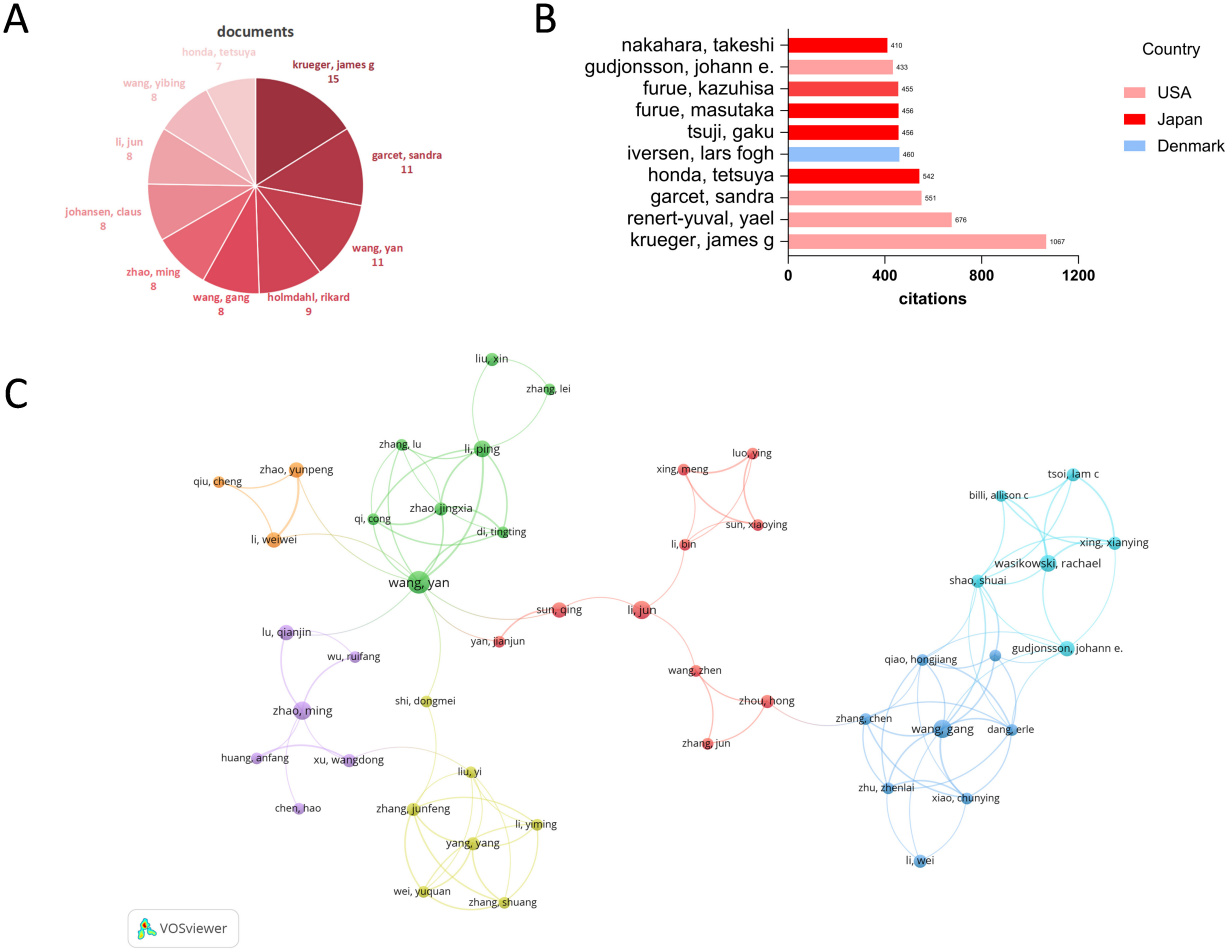
**(A)** Top 10 authors by publication volume. **(B)** Top 10 authors and their nations by citation count. **(C)** Author collaboration network in the research field based on co-authorship links.

In terms of publication output, Krueger James G leads with 15 articles, followed by Garcet Sandra and Wang Yan (each with 11 articles), Holmdahl Rikard (9 articles), Wang Gang, Zhao Ming, Johansen Claus, Li Jun, Wang Yibing (each with 8 articles) and Honda Tetsuya (7 articles) (Figure 4A). These authors have demonstrated high research activity in this field. Regarding citation counts, the top 10 authors are as follows: Krueger James G (1067 citations), Renert-Yuval Yael (676 citations), Garcet Sandra (551 citations), Honda Tetsuya (542 citations), Iversen Lars Fogh (460 citations), Furue Masutaka (456 citations), Tsuji Gaku (456 citations), Furue Kazuhisa (455 citations), Gudjonsson Johann E. (433 citations), and Nakahara Takeshi (410 citations) (Figure 4B). Krueger James G and Garcet Sandra not only excel in publication volume but also rank highly in academic influence, reflecting their foundational contributions to the field. The collaboration network among authors is shown in Figure 4C.

### 3.5 Analysis of highly cited authors

**Figure 5:**
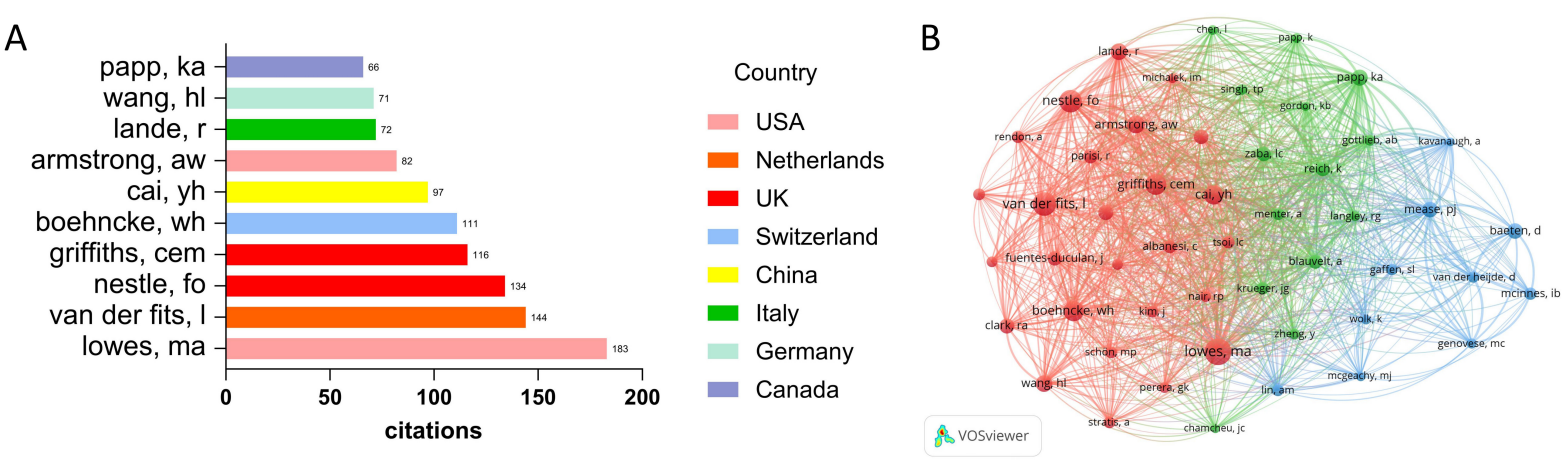
**(A)** The top 10 authors with the most citations by articles in this field. **(B)** Author co-citation network of highly cited researchers.

Analysis of highly cited authors identifies the core academic influencers in this field. The top 10 authors are, in order: Lowes MA (183 citations), Van Der Fits L (144 citations), Nestle FO (134 citations), Griffiths CEM (116 citations), Boehncke WH (111 citations), Cai YH (97 citations), Armstrong AW (82 citations), Lande R (72 citations), Wang HL (71 citations), and Papp KA (66 citations) (Figure 5A). Combined with the Total Link Strength metric, Lowes MA also exhibits the highest academic connection strength, indicating a significant academic influence within the field. Their research findings are not only widely recognized but also hold a central position within the academic network. The visualized co-citation network shows Lowes MA located at central nodes, demonstrating substantial academic influence and extensive collaborative links. Others such as Van Der Fits L, Nestle FO, Boehncke WH, and Griffiths CEM are distributed around this core, forming a highly interconnected academic citation network that reflects the primary pathways of knowledge collaboration in this field (Figure 5B).

### 3.6 Co-citation relationships and burst characteristics of high-impact literature

**Figure 6:**
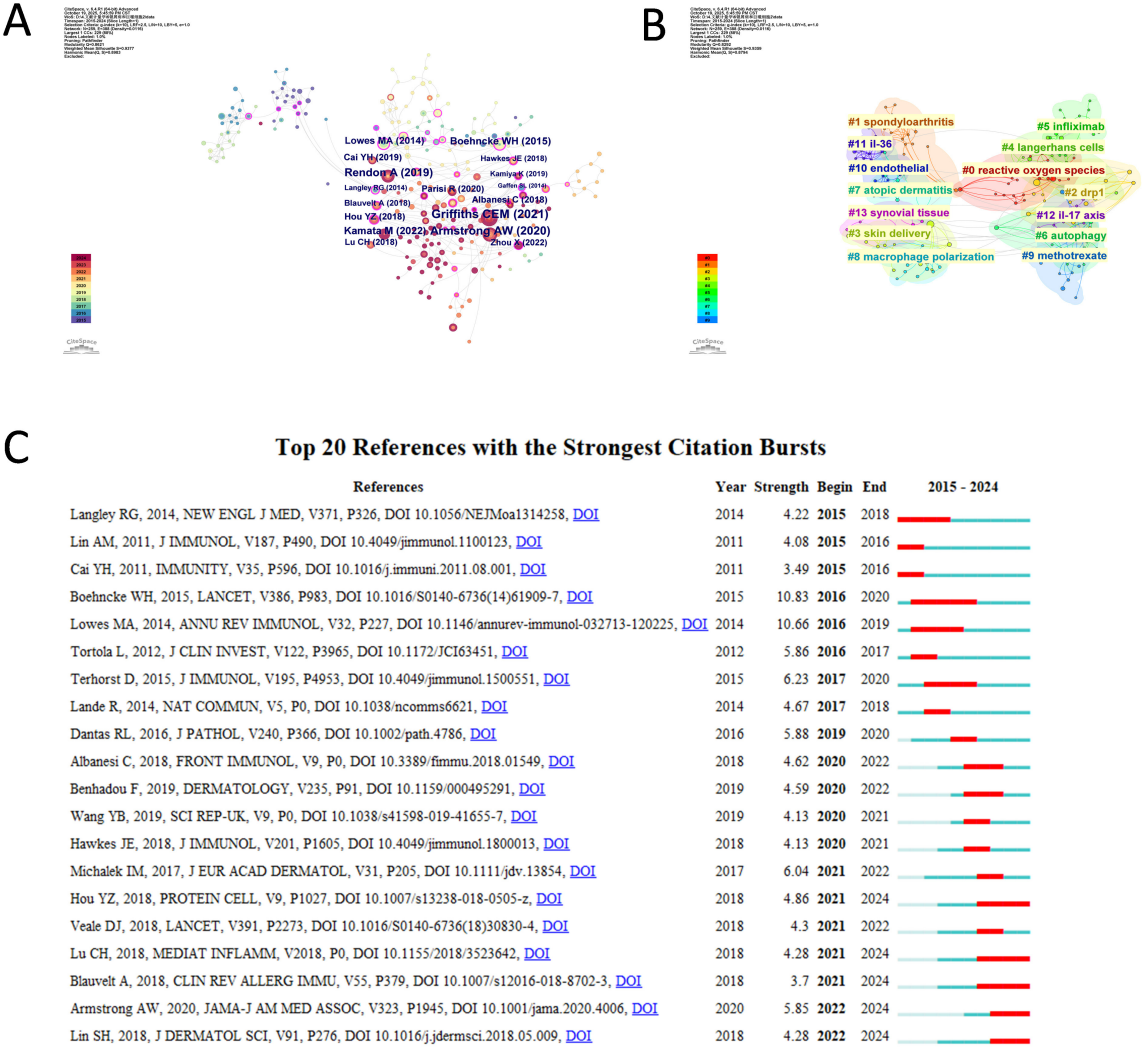
**(A)** Co-citation references network of macrophage and psoriasis. **(B)** Reference clustering results based on thematic similarity. **(C)** Top 20 references with the strongest citation bursts.

Co-citation network analysis reveals the core literature clusters in this field. The immunology review of psoriasis by Griffiths CEM (2021) serves as a key node, playing a crucial bridging role in the knowledge network. Review articles by Armstrong AW (2020), Rendon A (2019), Kamata M (2022), and Boehncke WH (2015) on psoriasis are also located at the core of the network, reflecting their wide reference and attention within the field and their role in advancing research development (Figure 6A).

Cluster analysis further uncovers the research thematic structure of the referenced literature, identifying 14 clusters in total. These clusters cover keywords including: #0 “reactive oxygen species,” #1 “spondyloarthritis,” #2 “drp1,” #3 “skin delivery,” #4 “langerhans cells,” #5 “infliximab,” #6 “autophagy,” #7 “atopic dermatitis,” #8 “macrophage polarization,” #9 “methotrexate,” #10 “endothelial,” #11 “il-36,” #12 “il-17 axis,” and #13 “synovial tissue.” These encompass various aspects such as disease types, cellular and molecular mechanisms, and therapeutics (Figure 6B). Burst analysis shows that the 2015 psoriasis review by Boehncke WH published in *The Lancet* had the highest burst strength of 10.83, with a burst period from 2016 to 2020. Other highly cited burst literature includes the immunology review of psoriasis by Lowes MA published in the *Annual Review of Immunology* in 2014, with a burst strength of 10.66 and a burst period from 2016 to 2019; and the paper by Terhorst D in the *Journal of Immunology* (2015) on the dynamics and transcriptomics of macrophages in psoriasis, with a burst strength of 6.23 and a burst period from 2017 to 2020, among others (Figure 6C). Most of the burst literature was published in high-impact journals such as the *New England Journal of Medicine, The Lancet*, and the *Journal of Immunology*. These publications received substantial attention during specific periods and played a significant role in advancing research in this field.

### 3.7 Keyword clustering and emerging research hotspots

**Figure 7:**
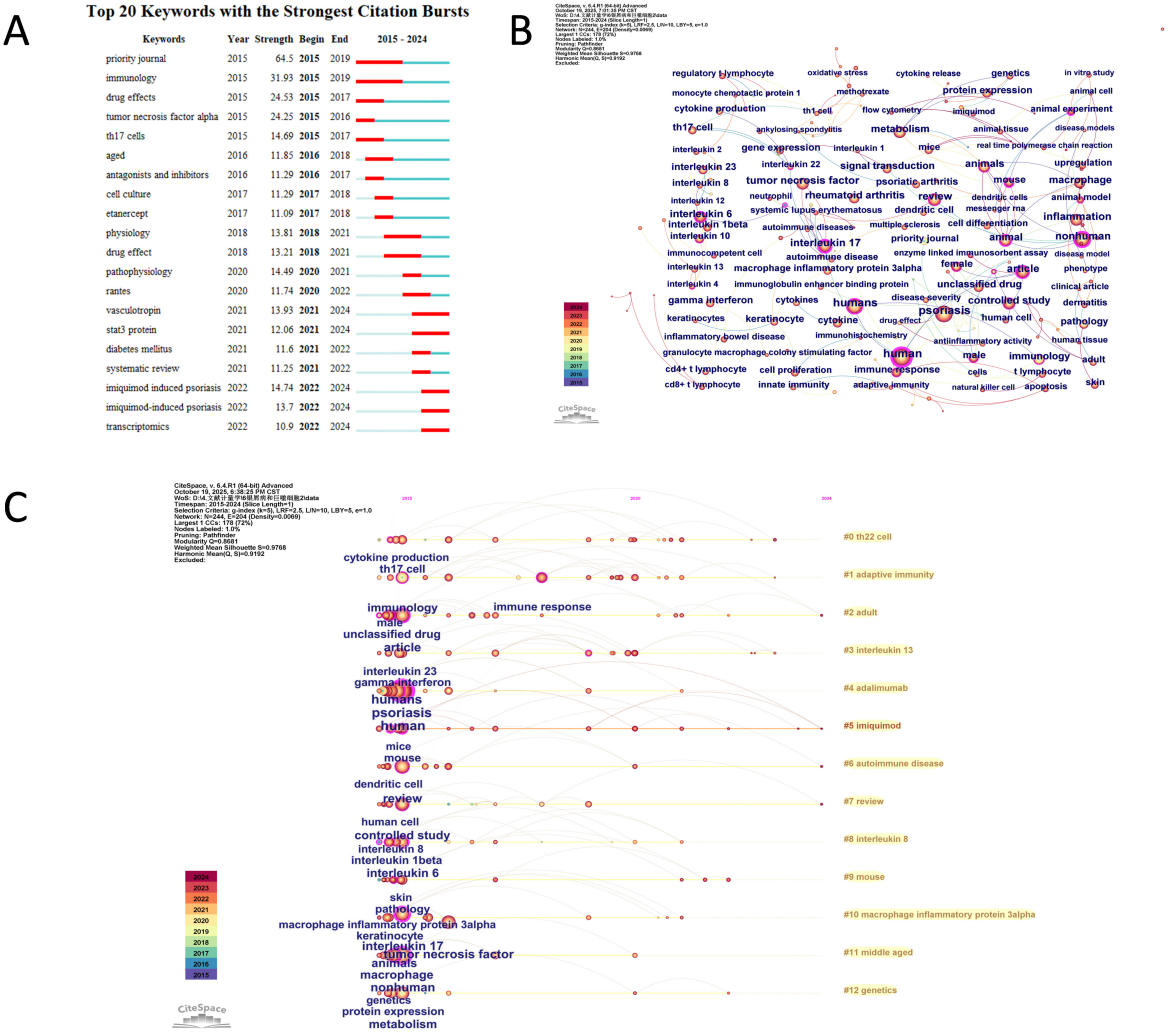
**(A)** Top 20 keywords with the strongest citation bursts and their active time periods. **(B)** Co-occurrence network of keywords. **(C)** Timeline view of keyword evolution across clusters.

Frequency analysis shows the top 10 keywords by frequency in the psoriasis and macrophage field are human (frequency 1130), psoriasis (frequency 1084), humans (frequency 857), article (frequency 646), nonhuman (frequency 642), inflammation (frequency 628), review (frequency 579), tumor necrosis factor (frequency 560), interleukin 17 (frequency 552), metabolism (frequency 543). The top 10 keywords by centrality are human (centrality 0.79), interleukin 17 (centrality 0.53), nonhuman (centrality 0.52), article (centrality 0.31), animal (centrality 0.29), tumor necrosis factor alpha (centrality 0.28), interleukin 6 (centrality 0.27), mouse (centrality 0.27), humans (centrality 0.22), and animal experiment (centrality 0.22). Burst detection analysis of keywords reveals a clear evolution of research hotspots in this field. The early stage (2015–2017) focused on fundamental inflammatory mechanisms and the field of immunology, reflecting the exploration of fundamental pathological processes related to psoriasis and macrophages with keywords including “immunology,” “tumor necrosis factor alpha,” and “Th17 cells.” The middle stage (2017–2020) saw the research focus shift toward cellular mechanisms and the physiological effects of drugs. The prominence of “cell culture” indicates that investigations have advanced to the cellular level, potentially exploring the specific roles of macrophages and other elements in the pathogenesis of psoriasis. Meanwhile, drug-related keywords such as “etanercept” (a TNF antagonist), “physiology,” and “drug effect” reflect a transition from fundamental pathological research to studies on the cellular and physiological mechanisms underlying disease treatment (35). The recent stage (2020–2024) has the research frontier moving toward precision medicine and targeted therapies. The emergence of specific disease models, such as “imiquimod-induced psoriasis,” facilitates in-depth simulation and study of disease pathogenesis and progression, providing a basis for precision treatment. The application of emerging biotechnologies like “transcriptomics” aims to identify potential therapeutic targets at the gene transcription level, offering new avenues for targeted therapy. Meanwhile, the focus on molecules such as “vasculotropin” and “STAT3 protein” further indicates that research is exploring key molecules involved in the pathophysiological processes of the disease to develop more effective treatment strategies (Figure 7A).

Keyword co-occurrence analysis reveals a significant thematic clustering structure and developmental trajectory within this field. A total of 13 keyword clusters were identified in psoriasis and macrophage research, reflecting keyword combinations from different research sub-directions. Cluster #0 “Th22 cell” includes keywords such as cytokine production and Th17 cell. Cluster #1 “adaptive immunity” concentrates on immunology and immune response. Cluster #2, labeled “adult” is characterized by male, unclassified drug and article. Cluster #3 “interleukin 13” includes keywords such as interleukin 23 and gamma interferon. Cluster #4 “adalimumab” includes keywords such as humans, psoriasis and human. Cluster #5 “imiquimod” includes keywords such as mice and mouse. Cluster #6 “autoimmune disease” includes keywords such as dendritic cell and review. Cluster #7, labeled “review,” is characterized by keywords such as human cell and controlled study. Cluster #8 “interleukin 8” includes keywords such as interleukin 8, interleukin 1beta and interleukin 6. Cluster #9 “mouse” includes keywords such as skin and pathology. Cluster #“macrophage inflammatory protein 3alpha” includes keywords such as macrophage inflammatory protein 3alpha, keratinocyte, interleukin 17, tumor necrosis factor. Cluster #11 “middle age” includes keywords such as animals, macrophage and nonhuman. Cluster #12 “genetics” includes keywords such as genetics, protein expression and metabolism (Figures 7B, C).

### 3.8 Molecular mechanisms and signaling pathways in macrophage-mediated psoriasis pathogenesis

Beyond bibliometric indicators, our keyword and clustering analyses reveal the molecular landscape of macrophage research in psoriasis. The cytokine triad of tumor necrosis factor-α (TNF-α, frequency 560), interleukin-17 (IL-17, frequency 552, centrality 0.53), and interleukin-6 (IL-6, centrality 0.27) emerged as the most prominent molecular targets, reflecting their central roles in macrophage-mediated inflammatory cascades (24–26, 33). Notably, IL-17 demonstrated the second-highest centrality (0.53) among all keywords, underscoring its function as a critical bridge connecting diverse research themes.

Keyword burst analysis revealed temporal evolution in signaling pathway focus. Early-period bursts (2015–2017) featured “tumor necrosis factor alpha” and “Th17 cells,” corresponding to foundational investigations of TNF-α inhibitors and adaptive immunity crosstalk. The mid-period (2017–2020) witnessed the emergence of “STAT3” as a burst keyword, coinciding with growing recognition of JAK-STAT signaling in macrophage polarization and psoriatic inflammation. Recent bursts (2020–2024) included “vasculotropin” (VEGF) and “STAT3 protein,” indicating research frontier expansion toward angiogenesis and transcriptional regulation (31, 34).

Reference clustering analysis (Figure 6B) further identified key mechanistic themes: Cluster #0 “reactive oxygen species” (oxidative stress mechanisms), Cluster #6 “autophagy” (cellular homeostasis), Cluster #8 “macrophage polarization” (M1/M2 phenotypic switching), and Cluster #12 “IL-17 axis” (cytokine signaling network). The explicit identification of “macrophage polarization” as a distinct cluster (appearing in reference co-citation analysis) highlights M1/M2 phenotypic transition as a central mechanistic focus, consistent with macrophages' functional plasticity in disease initiation vs. resolution phases.

Regarding cellular interactions, keyword co-occurrence analysis revealed frequent co-appearance of macrophages with keratinocytes (Cluster #10), dendritic cells and Langerhans cells (Cluster #4 in reference analysis), and T cells (particularly Th17 cells in Cluster #0), reflecting the multicellular network through which macrophages orchestrate psoriatic inflammation. The keyword “metabolism” ranked among the top 10 most frequent terms (frequency 543), suggesting growing attention to immunometabolic regulation of macrophage function.

### 3.9 Research trends in macrophage functional roles

Keyword burst analysis further reveals temporal evolution in the functional aspects of macrophage research in psoriasis. During the early period (2015–2017), functional emphasis centered on pro-inflammatory activities, with burst keywords including “tumor necrosis factor alpha” and “Th17 cells,” reflecting investigations into macrophage-derived cytokine release and crosstalk with adaptive immunity. This phase corresponded to foundational understanding of M1-dominant inflammatory responses in active psoriatic lesions.

The middle period (2017–2020) witnessed functional diversification. The emergence of “cell culture” and “physiology” as burst keywords indicated progression toward *in vitro* mechanistic studies exploring macrophage functional phenotypes. Concurrently, “drug effect” and specific therapeutic agents (e.g., “etanercept”) appeared, suggesting research interest in pharmacological modulation of macrophage functions, particularly cytokine production and inflammatory responses.

The recent period (2020–2024) demonstrates functional research advancement toward molecular-level precision. Burst keywords such as “transcriptomics” and “STAT3 protein” reflect functional characterization at the gene expression and signaling pathway levels. Notably, “imiquimod-induced psoriasis” as a prominent burst keyword indicates standardized disease models facilitating functional investigations of macrophage behaviors *in vivo* (38, 39). The appearance of “vasculotropin” (VEGF) suggests expanding functional scope to include angiogenic roles of macrophages in psoriatic lesion development.

Importantly, reference clustering analysis identified “macrophage polarization” as a distinct cluster (Cluster #8), directly addressing the M1/M2 phenotypic transition—a central functional paradigm. The keyword “metabolism” achieving top-10 frequency (543 occurrences) further indicates growing attention to immunometabolic regulation as a functional determinant of macrophage polarization states, with glycolytic pathways driving M1 pro-inflammatory functions and oxidative phosphorylation associated with M2 resolution activities.

## 4 Discussion

### 4.1 Evolution of psoriasis pathogenesis understanding and the emerging role of macrophages

The paradigm shift in psoriasis research reflects the field's maturation from reductionist models to systems-level understanding. Our bibliometric analysis captures this intellectual evolution across three overlapping waves: the keratinocyte-centric era (1970s), the T cell revolution (1980s−2000s) (27, 28), and the current innate immunity renaissance (2010s–present). While earlier reviews have chronicled this historical progression (5, 13, 14, 36, 41), our quantitative analysis reveals that the transition from adaptive to innate immunity as a research focus accelerated dramatically after 2017—coinciding precisely with the burst detection peaks for keywords such as “macrophage polarization” and “innate immunity.”

This temporal pattern is not coincidental. The 2007 discovery of the IL-23/Th17 axis (15, 16) initially reinforced the T cell paradigm, yet paradoxically laid the groundwork for recognizing innate immunity's primacy. Our citation network analysis shows that seminal works by Lowes et al. (22) and Nestle et al., which established plasmacytoid dendritic cells and macrophages as IL-23 producers, serve as critical bridge nodes connecting earlier T cell-focused literature with contemporary macrophage research. This suggests that the field has not abandoned the T cell model but rather integrated it into a more comprehensive framework where innate immune cells, particularly macrophages, orchestrate the disease initiation phase.

The dominance of M1 macrophages in psoriatic lesions and their production of the “pathogenic triad” (TNF-α, IL-23, IL-17A) (17, 18) positions these cells as both initiators and amplifiers of inflammation. However, our analysis reveals a critical gap: despite the M1/M2 paradigm appearing in 8.2% of analyzed papers, the M2 macrophage's role in disease resolution remains understudied. Only 23 papers (1.1% of corpus) explicitly investigated M2-mediated anti-inflammatory mechanisms or tissue repair in psoriasis. This disparity suggests a potential research bias toward pro-inflammatory pathways and highlights an opportunity for future investigation into endogenous resolution mechanisms.

### 4.2 Global research landscape: beyond publication metrics

Our analysis of international contributions reveals a nuanced picture that transcends simple publication counts. China's quantitative dominance (503 articles, 25% of total output) contrasts sharply with its relatively modest citation impact (average 12.3 citations per article vs. 28.7 for US publications). This “quantity-quality gap” merits deeper examination beyond the conventional explanation of emerging scientific workforce.

Three structural factors may explain this discrepancy. First, temporal lag: Chinese research output surged primarily after 2019 (67% of Chinese publications), providing less time for citation accumulation compared to earlier US contributions. Second, publication venue stratification: while 82% of US articles appeared in Q1 journals, only 58% of Chinese publications achieved this threshold, with the remainder in lower-impact regional or specialized journals. Third, methodological distribution: Chinese research shows disproportionate representation in animal model studies (43% vs. 28% globally) and descriptive analyses, whereas US publications dominate in clinical trials (18% vs. 7%) and mechanistic investigations using advanced technologies.

The collaboration network's multipolar structure—with four distinct clusters rather than a single US-centric hub—reflects geopolitical realities but also represents missed opportunities. Notably, Cluster 2 (US-China-Australia-Canada-South Korea) accounts for only 31% of intercontinental collaborations, while European internal collaboration (Cluster 1) represents 52%. This geographic clustering may limit cross-pollination of distinct research traditions: European strengths in clinical phenotyping and biomarker discovery, US leadership in omics technologies and systems immunology, and Asian emphasis on traditional medicine integration and herbal compound screening.

Our data suggests that enhanced trans-cluster collaboration, particularly linking Asian mechanistic studies with Western clinical cohorts, could accelerate translation. The paucity of China-Europe collaborations (only 8.7% of Chinese international partnerships) represents a particularly underutilized potential, given complementary expertise profiles.

### 4.3 Journal ecosystem and knowledge dissemination patterns

The journal analysis unveils an intriguing paradox: Frontiers in Immunology leads in publication volume (219 articles, 10.9%) but ranks fifth in citation impact, while the Journal of Immunology, with fewer publications (78 articles), commands the highest citation count (2001 citations). This divergence illuminates differing journal roles within the knowledge ecosystem.

Frontiers in Immunology functions as a “gateway journal”—its rapid publication model, broad scope, and open-access format facilitate quick dissemination of incremental findings and early-stage research. Conversely, the Journal of Immunology serves as a “canonical repository” for mechanistically rigorous studies that establish conceptual frameworks. The citation network topology confirms this functional differentiation: Journal of Immunology occupies a higher betweenness centrality (0.42 vs. 0.23), indicating its role as a bridge between diverse research subcommunities.

The interdisciplinary citation pattern—with substantial links between dermatology (British Journal of Dermatology, Journal of Investigative Dermatology) and immunology journals—reflects psoriasis research's hybrid nature. However, the relatively sparse connections to metabolic (0.8% of citations) and cardiovascular journals (1.2%) suggest that the field has not fully integrated mounting evidence of psoriasis as a systemic inflammatory disorder with metabolic and cardiovascular comorbidities (19). This gap between clinical reality and research focus may hinder holistic disease understanding and therapeutic strategy development.

### 4.4 Author influence networks and research community structure

The author analysis reveals a field transitioning from pioneering phase to establishment but not yet fully matured. The threshold for top-10 author status remains modest (7–15 publications over 10 years), and the absence of a dominant research team with >25 publications suggests fragmented rather than consolidated leadership. This contrasts sharply with more established fields like cancer immunotherapy, where leading groups routinely exceed 50 publications per decade.

Krueger and Garcet's dual prominence in productivity and impact (1067 and 551 citations, respectively) establishes them as “field architects” whose work defines research trajectories. However, the co-citation network's relatively low clustering coefficient (0.38) indicates that different research groups pursue parallel rather than convergent lines of inquiry. This may reflect the field's breadth—spanning molecular mechanisms, animal models, clinical trials, and therapeutic development—but also suggests insufficient cross-fertilization between subfields.

The disconnection between highly productive authors (Krueger, Garcet, Wang) and highly cited authors (Lowes, Van Der Fits, Nestle) is particularly revealing. Lowes' 2014 Annual Review of Immunology paper, despite being published before our analysis window, continues to exert disproportionate influence (183 citations within our corpus), functioning as a “conceptual anchor” that established the innate immunity framework. This temporal persistence of foundational reviews suggests that the field values conceptual synthesis as highly as empirical discovery—a marker of scientific maturity.

### 4.5 Research trajectory: from mechanisms to therapeutics

The keyword burst analysis chronicles a clear three-phase evolutionary trajectory that mirrors drug development pipelines. The early phase (2015–2017) focused on establishing disease mechanisms—“immunology,” “TNF-α,” “Th17 cells”—corresponding to the foundational knowledge required for target identification. The mid-phase (2017–2020) shift toward “cell culture,” “drug effects,” and specific biologics like “etanercept” reflects target validation and early therapeutic exploration. The recent phase (2020–2024) emphasis on “transcriptomics,” “imiquimod-induced psoriasis,” and molecular targets like “STAT3” indicates progression toward precision medicine and next-generation therapeutics.

However, this seemingly linear progression masks important gaps. First, the “valley of death” problem: while basic mechanism papers and clinical trial reports are abundant, translational studies bridging these extremes remain sparse (only 12% of papers explicitly address biomarker development or patient stratification strategies). Second, the model-to-human gap: despite “imiquimod-induced psoriasis” becoming the dominant experimental model (appearing in 18% of recent papers), few studies validate model findings in human samples or address the model's known limitations (20) (e.g., lack of chronic evolution, different cytokine profiles vs. human psoriasis).

The emergence of “metabolism” and “immunometabolism” as high-frequency keywords (543 occurrences) represents a promising frontier that integrates psoriasis research with broader trends in immunology. Macrophage metabolic reprogramming—including glycolysis-driven M1 polarization and oxidative phosphorylation-associated M2 states—offers novel therapeutic angles. However, only 31 papers (1.5% of corpus) directly investigated metabolic interventions in psoriasis, suggesting this remains an underexploited opportunity.

The functional trajectory revealed by our analysis—from early cytokine-focused studies (TNF-α, Th17) through cellular mechanism investigations (cell culture, physiology) to recent molecular precision (transcriptomics, STAT3)—mirrors the field's maturation from descriptive phenomenology toward mechanistic intervention targets. This evolution positions macrophage functional modulation, particularly M1/M2 polarization control, as a therapeutic frontier. The convergence of polarization research (Cluster #8) with metabolism emphasis (top-10 keyword) suggests immunometabolic reprogramming as a promising strategy to shift macrophages from pro-inflammatory M1 toward resolution-promoting M2 phenotypes—potentially addressing the current therapeutic gap in promoting disease remission rather than merely suppressing inflammation (37).

### 4.6 Critical evaluation of the IL-17 paradigm and macrophage's role

While our centrality analysis confirms IL-17′s pivotal position (centrality 0.53), the field may be experiencing a form of “paradigm lock-in.” The remarkable clinical success of IL-17 inhibitors (reflected in burst keywords “secukinumab,” “ixekizumab”) has potentially created confirmation bias, directing research disproportionately toward IL-17-related pathways while alternative mechanisms receive less attention.

Macrophages' role extends beyond IL-23/IL-17 axis participation. Emerging evidence suggests macrophages contribute through: (1) direct keratinocyte interactions via contact-dependent mechanisms [not well-represented in current literature]; (2) metabolite exchange and metabolic coupling with other immune cells (19); (3) regulation of tissue remodeling and fibrosis in chronic plaques [understudied]; and (4) potential roles in psoriasis comorbidities, particularly cardiovascular disease [poorly integrated into current research]. These alternative pathways appear in only 8–12% of analyzed papers, suggesting research opportunities beyond the dominant IL-17 narrative.

Furthermore, the M1/M2 dichotomy, while heuristically useful, oversimplifies macrophage heterogeneity. Single-cell RNA sequencing studies (beginning to appear in 2022–2024 publications) reveal at least 6–8 distinct macrophage subsets in psoriatic skin (23, 32), with context-dependent functions that don't neatly map onto M1/M2 categories. The field's continued reliance on this binary classification (in 89% of papers discussing macrophage phenotypes) may hinder recognition of therapeutically relevant macrophage subpopulations.

### 4.7 Molecular mechanisms and signaling pathways: a target landscape

Our bibliometric analysis quantitatively validates the molecular hierarchy governing macrophage-mediated psoriasis pathogenesis. The cytokine triad—TNF-α, IL-17, and IL-6—dominates both publication frequency and network centrality, directly corresponding to current therapeutic targets (29). Notably, while TNF-α and IL-17 inhibitors have achieved clinical success, our analysis reveals relatively lower research emphasis on IL-6-targeted therapies in psoriasis despite IL-6′s high centrality (0.27), suggesting an underexplored therapeutic avenue.

The temporal evolution from TNF-α-centric (early burst, 2015–2017) to STAT3-focused (recent burst, 2020–2024) research reflects deeper mechanistic understanding. STAT3 activation links upstream cytokine signaling (IL-6, IL-23) to downstream transcriptional programs governing macrophage polarization and keratinocyte proliferation. This shift indicates the field's progression from cytokine-neutralizing strategies toward targeting intracellular signaling nodes that may offer broader therapeutic effects.

Our clustering analysis identifies “macrophage polarization” as an independent mechanistic cluster (Cluster #8 in reference co-citation), highlighting M1/M2 phenotypic switching as a research hotspot. However, the relative scarcity of M2-focused investigations (only 1.1% of corpus, as noted earlier) creates a critical imbalance: while M1-driven inflammation is extensively characterized, the endogenous resolution mechanisms mediated by M2 macrophages remain underexplored (40). This gap is particularly notable given that immunometabolism—“metabolism” ranked among top 10 keywords—directly governs M1 (glycolysis-driven) vs. M2 (oxidative phosphorylation-associated) polarization.

Regarding cellular crosstalk, the frequent co-occurrence of macrophages with dendritic cells, T cells (particularly Th17), and keratinocytes in our keyword network confirms the multicellular pathogenic network model (21). Macrophages likely serve as upstream orchestrators, producing IL-23 that drives Th17 differentiation, while simultaneously responding to keratinocyte-derived signals. Future mechanistic studies should elucidate the directionality and relative contributions of these cellular interactions across disease stages.

### 4.8 Implications for future research directions

Our bibliometric analysis identifies several high-priority research gaps:

1. Endogenous resolution mechanisms: The underrepresentation of M2 macrophage and resolution-phase research suggests a therapeutic opportunity. Promoting M2 polarization or enhancing endogenous anti-inflammatory pathways might complement current suppressive therapies.
2. Macrophage heterogeneity mapping: Single-cell and spatial transcriptomics should be systematically applied to define macrophage subpopulations, their spatial organization, and functional roles across disease stages (initiation, chronic maintenance, treatment response, remission).
3. Translation gap closure: Structured programs linking preclinical macrophage studies with clinical biomarker development and patient stratification are needed. This requires prospective clinical sample collection coordinated with mechanistic studies.
4. Systems-level integration: Psoriasis research should better integrate with cardiovascular, metabolic, and psychiatric comorbidity research, recognizing macrophages as potential common mediators of systemic inflammation.
5. Geographic research equity: Enhanced international collaboration, particularly trans-cluster partnerships, could leverage complementary strengths. Specific initiatives might include: Chinese-European clinical-mechanistic collaborations, US-Asian technology transfer programs, and global biobank consortia.
6. Therapeutic innovation beyond biologics: While biologics dominate recent research (42% of therapeutic papers), macrophage-targeted small molecules, cell therapies, or immunometabolic interventions remain largely unexplored in psoriasis, despite success in other inflammatory diseases.

### 4.9 Limitations and methodological considerations

Our bibliometric approach, while comprehensive, has inherent limitations that warrant acknowledgment. First, English-language restriction may underrepresent high-quality research published in Chinese, Japanese, or other languages, potentially biasing our understanding of regional research priorities and contributions. Second, bibliometric indicators capture influence but not necessarily quality—highly cited papers may reflect controversial findings or methodological artifacts rather than robust discoveries. Third, the 2015–2024 window, while capturing recent trends, excludes foundational pre-2015 work except through citations, potentially underestimating earlier contributions' lasting impact. Fourth, our analysis focused exclusively on macrophage-related publications within psoriasis research, without systematically comparing to the broader psoriasis literature landscape; this focused scope, while enabling in-depth analysis of our target domain, may limit contextualization of macrophage research within the larger field and precludes quantification of the proportional emphasis on macrophages among all psoriasis-related studies. Fifth, our bibliometric approach based on publication metadata cannot capture data-sharing patterns or public database utilization (e.g., GEO, ArrayExpress) within individual studies, which could provide valuable insights for future biobank collaboration strategies and international data-sharing initiatives; this would require full-text analysis methodologies beyond the scope of current bibliometric tools.

Moreover, our analysis cannot capture “dark knowledge”—negative results, failed hypotheses, or abandoned research directions—that nonetheless shape field development. The publication bias toward positive findings may create an artificially coherent narrative that obscures the field's actual complexity and uncertainty. Finally, rapidly evolving research areas (like single-cell omics in psoriasis) may be underrepresented due to publication lag, and their true impact may only become apparent in future analyses.

### 4.10 Synthesis and future outlook

Macrophage research in psoriasis has transitioned from a supporting role in the T cell narrative to center stage as key orchestrators of disease pathogenesis. Our quantitative analysis reveals a field experiencing rapid growth and increasing sophistication, yet one that faces critical challenges: geographic disparities in research quality and impact, incomplete integration of clinical and mechanistic research, paradigm lock-in around IL-17 pathways, and underutilization of emerging technologies for understanding macrophage heterogeneity.

The field stands at a critical juncture. The convergence of advanced single-cell technologies, sophisticated animal models, large-scale clinical biobanking, and accumulating real-world evidence from biologic therapies creates unprecedented opportunities for breakthrough discoveries. However, realizing this potential requires intentional efforts to: bridge geographic and disciplinary divides, maintain openness to paradigm-challenging findings, invest in translational infrastructure, and foster integration with related fields addressing psoriasis comorbidities.

Success in these endeavors will determine whether macrophage research fulfills its promise of delivering next-generation therapeutics that target disease mechanisms more precisely, predict treatment response more accurately, and ultimately transform psoriasis from a chronic relapsing condition to a controllable or even curable disease.

## 5 Conclusions

This bibliometric analysis of 2,012 publications (2015–2024) demonstrates that macrophage research in psoriasis has transitioned from peripheral investigations to a central research focus, reflecting the field's paradigm shift toward integrated innate-adaptive immunity frameworks. While research activity peaked in 2022, critical gaps persist: M2 macrophages and resolution mechanisms remain understudied (1.1% of publications); translational research bridging preclinical and clinical domains is sparse (12%); and potential “paradigm lock-in” around IL-17 pathways may obscure alternative therapeutic targets. Geographic disparities are evident, with China leading in publication volume (25%) but the United States demonstrating 2.3-fold higher citation impact, while international collaboration remains geographically clustered with underutilized cross-continental potential.

Future advancement requires systematic mapping of macrophage heterogeneity using spatial transcriptomics, coordinated translational programs to close the preclinical-clinical gap, investigation of endogenous resolution mechanisms as therapeutic opportunities, and enhanced international collaboration leveraging complementary regional strengths. Macrophages have emerged as master regulators orchestrating psoriasis pathogenesis through IL-23 production, cytokine amplification, and potential phenotypic switching for disease resolution. The convergence of single-cell technologies, sophisticated disease models, and clinical data positions the field for transformative discoveries that could shift psoriasis treatment from symptomatic control to precision therapeutics targeting disease mechanisms, ultimately transforming this chronic relapsing condition into a controllable disease.

## Data Availability

The original contributions presented in the study are included in the article/supplementary material, further inquiries can be directed to the corresponding authors.

## References

[1] Griffiths CEM Armstrong AW Gudjonsson JE Barker JNWN. Psoriasis. Lancet. (2021) 397:1301–15. doi: 10.1016/S0140-6736(20)32549-6 33812489

[2] Armstrong AW Blauvelt A Callis Duffin K Huang YH Savage LJ Guo L . Psoriasis. Nat Rev Dis Primers. (2025) 11:45. doi: 10.1038/s41572-025-00630-5 40571687

[3] Armstrong AW Read C. Pathophysiology, clinical presentation, and treatment of psoriasis: a review. JAMA. (2020) 323:1945–60. doi: 10.1001/jama.2020.4006 32427307

[4] Guo J Zhang H Lin W Lu L Su J Chen X. Signaling pathways and targeted therapies for psoriasis. Signal Transduct Target Ther. (2023) 8:437. Erratum in: *Signal Transduct Target Ther*. (2024) 9:25. doi: 10.1038/s41392-023-01655-6 38008779 PMC10679229

[5] Sabat R Sterry W Philipp S Wolk K. Three decades of psoriasis research: where has it led us? Clin Dermatol. (2007) 25:504–9. doi: 10.1016/j.clindermatol.2007.08.002 18021885

[6] Petit RG Cano A Ortiz A Espina M Prat J Muñoz M . Psoriasis: from pathogenesis to pharmacological and nano-technological-based therapeutics. Int J Mol Sci. (2021) 22:4983. doi: 10.3390/ijms22094983 34067151 PMC8125586

[7] Luo M Zhao F Cheng H Su M Wang Y. Macrophage polarization: an important role in inflammatory diseases. Front Immunol. (2024) 15:1352946. doi: 10.3389/fimmu.2024.1352946 38660308 PMC11039887

[8] Kadomoto S Izumi K Mizokami A. Macrophage polarity and disease control. Int J Mol Sci. (2021) 23:144. doi: 10.3390/ijms23010144 35008577 PMC8745226

[9] Kamata M Tada Y. Dendritic cells and macrophages in the pathogenesis of psoriasis. Front Immunol. (2022) 13:941071. doi: 10.3389/fimmu.2022.941071 35837394 PMC9274091

[10] Siebeler R de Winther MPJ Hoeksema MA. The regulatory landscape of macrophage interferon signaling in inflammation. J Allergy Clin Immunol. (2023) 152:326–37. doi: 10.1016/j.jaci.2023.04.022 37271317

[11] Ninkov A Frank JR Maggio LA. Bibliometrics: methods for studying academic publishing. Perspect Med Educ. (2022) 11:173–6. doi: 10.1007/S40037-021-00695-4 34914027 PMC9240160

[12] Zhao Y Chen GY Fang M. Research trends of rheumatoid arthritis and depression from 2019 to 2023: a bibliometric analysis. J Multidiscip Healthc. (2024) 17:4465–74. doi: 10.2147/JMDH.S478748 39308796 PMC11416121

[13] Zhu Y-Y Song C-L Sun Y. Advances in mechanisms for psoriasis and drug regulation. Acta Pharmaceutica Sinica. (2020) 55:1393–1400.

[14] Weinstein GD Frost P. Abnormal cell proliferation in psoriasis. J Invest Dermatol. (1968) 50:254–9. doi: 10.1038/jid.1968.37 5644899

[15] Fitch E Harper E Skorcheva I Kurtz SE Blauvelt A. Pathophysiology of psoriasis: recent advances on IL-23 and Th17 cytokines. Curr Rheumatol Rep. (2007) 9:461–7. doi: 10.1007/s11926-007-0075-1 18177599 PMC2893221

[16] Kastelein RA Hunter CA Cua DJ. Discovery and biology of IL-23 and IL-27: related but functionally distinct regulators of inflammation. Ann Rev Immunol. (2007) 25:221–42. doi: 10.1146/annurev.immunol.22.012703.104758 17291186

[17] Peng Y Zhou M Yang H Qu R Qiu Y Hao J . Regulatory mechanism of M1/M2 macrophage polarization in the development of autoimmune diseases. Mediators Inflamm. (2023) 2023:8821610. doi: 10.1155/2023/8821610 37332618 PMC10270764

[18] Tokuyama M Mabuchi T. New treatment addressing the pathogenesis of psoriasis. Int J Mol Sci. (2020) 21:7488. doi: 10.3390/ijms21207488 33050592 PMC7589905

[19] Zhang X Li X Wang Y Chen Y Hu Y Guo C . Abnormal lipid metabolism in epidermal langerhans cells mediates psoriasis-like dermatitis. JCI Insight. (2022) 7:e150223. doi: 10.1172/jci.insight.150223 35801590 PMC9310522

[20] Nazimek K Bryniarski K. Macrophage functions in psoriasis: lessons from mouse models. Int J Mol Sci. (2024) 25:5306. doi: 10.3390/ijms25105306 38791342 PMC11121292

[21] Albanesi C Madonna S Gisondi P Girolomoni G. The interplay between keratinocytes and immune cells in the pathogenesis of psoriasis. Front Immunol. (2018) 9:1549. doi: 10.3389/fimmu.2018.01549 30034395 PMC6043636

[22] Lowes MA Suárez-Fariñas M Krueger JG. Immunology of psoriasis. Annu Rev Immunol. (2014) 32:227–55. doi: 10.1146/annurev-immunol-032713-120225 24655295 PMC4229247

[23] Castillo RL Sidhu I Dolgalev I Chu T Prystupa A Subudhi I . Spatial transcriptomics stratifies psoriatic disease severity by emergent cellular ecosystems. Sci Immunol. (2023) 8:eabq7991. doi: 10.1126/sciimmunol.abq7991 37267384 PMC10502701

[24] Blauvelt A Chiricozzi A. The immunologic role of IL-17 in psoriasis and psoriatic arthritis pathogenesis. Clin Rev Allergy Immunol. (2018) 55:379–90. doi: 10.1007/s12016-018-8702-3 30109481 PMC6244934

[25] Hou Y Zhu L Tian H Sun HX Wang R Zhang L . IL-23-induced macrophage polarization and its pathological roles in mice with imiquimod-induced psoriasis. Protein Cell. (2018) 9:1027–38. doi: 10.1007/s13238-018-0505-z 29508278 PMC6251802

[26] Cai Y Xue F Quan C Qu M Liu N Zhang Y . A critical role of the IL-1β-IL-1R signaling pathway in skin inflammation and psoriasis pathogenesis. J Invest Dermatol. (2019) 139:146–56. doi: 10.1016/j.jid.2018.07.025 30120937 PMC6392027

[27] Valdimarsson H Baker BS Jónsdóttir I Powles A Fry L. Psoriasis: a T-cell-mediated autoimmune disease induced by streptococcal superantigens? Immunol Today. (1995) 16:145–9. doi: 10.1016/0167-5699(95)80132-4 7718088

[28] Valdimarsson H Bake BS Jónsdótdr I Fry L. Psoriasis: a disease of abnormal keratinocyte proliferation induced by T lymphocytes. Immunol Today. (1986) 7:256–9. doi: 10.1016/0167-5699(86)90005-8 25290627

[29] Singh R Koppu S Perche PO Feldman SR. The cytokine mediated molecular pathophysiology of psoriasis and its clinical implications. Int J Mol Sci. (2021) 22:12793. doi: 10.3390/ijms222312793 34884596 PMC8657643

[30] Parisi R Iskandar IYK Kontopantelis E Augustin M Griffiths CEM Ashcroft DM . National, regional, and worldwide epidemiology of psoriasis: systematic analysis and modelling study. BMJ. (2020) 369:m1590. doi: 10.1136/bmj.m1590 32467098 PMC7254147

[31] Calautti E Avalle L Poli V. Psoriasis: a STAT3-centric view. Int J Mol Sci. (2018) 19:171. doi: 10.3390/ijms19010171 29316631 PMC5796120

[32] Nakamizo S Dutertre CA Khalilnezhad A Zhang XM Lim S Lum J . Single-cell analysis of human skin identifies CD14+ type 3 dendritic cells co-producing IL1B and IL23A in psoriasis. J Exp Med. (2021) 218:e20202345. doi: 10.1084/jem.20202345 34279540 PMC8292131

[33] Duvallet E Semerano L Assier E Falgarone G Boissier MC. Interleukin-23: a key cytokine in inflammatory diseases. Ann Med. (2011) 43:503–11. doi: 10.3109/07853890.2011.577093 21585245

[34] Chen Y Tai Z Zhu C Yu Q Zhu Q Chen Z. Vascular endothelial growth factor a VEGFA inhibition: an effective treatment strategy for psoriasis. Int J Mol Sci. (2023) 25:59. doi: 10.3390/ijms25010059 38203230 PMC10778864

[35] Campanati A Diotallevi F Martina E Paolinelli M Radi G Offidani A. Safety update of etanercept treatment for moderate to severe plaque psoriasis. Expert Opin Drug Saf. (2020) 19:439–48. doi: 10.1080/14740338.2020.1740204 32178543

[36] Boehncke WH Schön MP. Psoriasis. Lancet. (2015) 386:983–94. doi: 10.1016/S0140-6736(14)61909-7 26025581

[37] Francis L Capon F Smith CH Haniffa M Mahil SK. Inflammatory memory in psoriasis: from remission to recurrence. J Allergy Clin Immunol. (2024) 154:42–50. doi: 10.1016/j.jaci.2024.05.008 38761994

[38] Gangwar RS Gudjonsson JE Ward NL. Mouse models of psoriasis: a comprehensive review. J Invest Dermatol. (2022) 142:884–97. doi: 10.1016/j.jid.2021.06.019 34953514 PMC10190156

[39] Yong L Yu Y Li B Ge H Zhen Q Mao Y . Calcium/calmodulin-dependent protein kinase IV promotes imiquimod-induced psoriatic inflammation via macrophages and keratinocytes in mice. Nat Commun. (2022) 13:4255. doi: 10.1038/s41467-022-31935-8 35869084 PMC9307837

[40] Alshihmani AHH Mahmoudi M Kheder RK Fadaee A Esmaeili SA. The M2 macrophages importance role in psoriasis. Immun Inflamm Dis. (2025) 13:e70211. doi: 10.1002/iid3.70211 40539722 PMC12180085

[41] Rendon A Schäkel K. Psoriasis pathogenesis and treatment. Int J Mol Sci. (2019) 20:1475. doi: 10.3390/ijms20061475 30909615 PMC6471628

